# Psychosocial factors associated with the mental health of indigenous children living in high income countries: a systematic review

**DOI:** 10.1186/s12939-017-0652-5

**Published:** 2017-08-23

**Authors:** Christian Young, Camilla Hanson, Jonathan C. Craig, Kathleen Clapham, Anna Williamson

**Affiliations:** 1 Sydney School of Public Health, The University of Sydney, Edward Ford Building (A27), Fisher Road, Camperdown, NSW 2006 Australia; 2 Centre for Kidney Research, Westmead Institute for Medical Research, 179 Hawkesbury Rd, Westmead, NSW 2145 Australia; 3 Australian Health Services Research Institute, Innovation Campus, University of Wollongong, Building 234 (iC Enterprise 1), Wollongong, NSW 2522 Australia; 4 The Sax Institute, Level 13, Building 10, 235 Jones Street, Ultimo, NSW 2007 Australia

**Keywords:** Indigenous, Children, Adolescent, Mental health, Psychosocial, Review

## Abstract

**Background:**

Indigenous children living in high income countries have a consistently high prevalence of mental health problems. We aimed to identify psychosocial risk and protective factors for mental health in this setting.

**Methods:**

A systematic review of studies published between 1996 and 2016 that quantitatively evaluated the association between psychosocial variables and mental health among Indigenous children living in high income countries was conducted. Psychosocial variables were grouped into commonly occurring domains. Individual studies were judged to provide evidence for an association between a domain and either good mental health, poor mental health, or a negligible or inconsistent association. The overall quality of evidence across all studies for each domain was assessed using the Grades of Recommendation, Assessment, Development, and Evaluation (GRADE) guidelines.

**Results:**

Forty-seven papers were eligible (mainland US 30 [64%], Canada 8 [17%], Australia 7 [15%], Hawaii 4 [9%]), including 58,218 participants aged 4–20 years. Most papers were cross-sectional (39, 83%) and measured negative mental health outcomes (41, 87%). Children’s negative cohesion with their families and the presence of adverse events appeared the most reliable predictors of increased negative mental health outcomes. Children’s substance use, experiences of discrimination, comorbid internalising symptoms, and negative parental behaviour also provided evidence of associations with negative mental health outcomes. Positive family and peer relationships, high self-esteem and optimism were associated with increased positive mental health outcomes.

**Conclusions:**

Quantitative research investigating Indigenous children’s mental health is largely cross-sectional and focused upon negative outcomes. Indigenous children living in high income countries share many of the same risk and protective factors associated with mental health. The evidence linking children’s familial environment, psychological traits, substance use and experiences of discrimination with mental health outcomes highlights key targets for more concerted efforts to develop initiatives to improve the mental health of Indigenous children.

**Electronic supplementary material:**

The online version of this article (doi:10.1186/s12939-017-0652-5) contains supplementary material, which is available to authorized users.

## Background

Indigenous children living in high income countries such as Australia, New Zealand, Canada and the United States (US) are disproportionately affected by mental health problems when compared to their non-Indigenous counterparts [1–5]. Childhood mental health disorders such as anxiety, depression and externalising behaviours are associated with a range of negative outcomes that are overrepresented in Indigenous communities, including high rates of suicidal ideation and completion [6, 7]. The long-term sequelae of poor childhood mental health is believed to significantly contribute to negative health and social outcomes that occur throughout the lifespan [8].

While the aetiology of childhood mental health disorders is likely to involve multiple determinants, the impact of European colonisation constitutes an additional, pervasive risk factor for Indigenous children living in Australia, New Zealand, Canada and the US. For these children, colonisation and subsequent cultural marginalisation are believed to be the “cause of causes” [9], impacting negatively on children’s mental health through low socio-economic families and communities, experiences of discrimination, and exposure to the psychological effects of intergenerational trauma and inequality [10].

Given that Indigenous populations share a history of colonisation, research that investigates common correlates of mental health may help to strengthen the evidence base, and contribute to the development of effective health interventions. To date, there has been little research that assesses risk and protective factors among multiple Indigenous cultures. The aim of this systematic review is to identify modifiable psychosocial risk and protective factors, common to Indigenous children living in Australia, New Zealand, Canada and the US that are associated with mental health outcomes typically experienced during childhood and adolescence. The results may aid the design of initiatives to improve the mental health of Indigenous children, reduce health disparities, and identify areas for further research.

## Methods

We followed the Meta-analysis of Observational Studies in Epidemiology (MOOSE) guidelines to conduct this systematic review [11].

### Study inclusion and exclusion criteria

Peer-reviewed, English language studies that reported quantified relationships between psychosocial variables and mental health outcomes in Indigenous children were eligible. School-aged samples (mean ages between 5 and 18 years) from the four ‘CANZUS’ (Canada, Australia, New Zealand, United States) countries were included, with studies including participants over 21 years excluded. Given differences in the environmental and social challenges Indigenous populations living within the Arctic Circle experience compared to other Indigenous communities, studies involving these populations were excluded [12]. Studies investigating multiple ethnic groups were included if a separate quantitative analysis was provided for the Indigenous sample.

Due to the potential of evolving social and political landscapes to effect changes in the health of Indigenous minority groups, only papers published in the last 20 years (1996 to January 2016) were included. In keeping with this review’s focus of modifiable factors associated with mental health, studies measuring congenital disorders or mental disability were excluded. Given current controversies surrounding the diagnosis of Attention Deficit Hyperactivity Disorder (ADHD) [13], associations between psychosocial variables and an ADHD diagnosis were not included.

Symptoms of mental health vary considerably in both presentation and severity. This review focused on commonly measured aspects of mental health that are relevant from early childhood to late adolescence and across a range of cultures. These included externalising and internalising disorders, and measures of positive mental health such as self-esteem [14]. In keeping with this focus, outcomes that were more serious, rare and less likely to be observed across the relevant age range such as eating disorders, delinquency and suicidal ideation and completion were excluded [15–18]. Studies that used recruitment strategies that led to over-sampling high risk populations were not included.

### Search strategy

The first author (CY) conducted the literature search using MEDLINE, PsychINFO, Embase, and Scopus databases. Results were retrieved in February, 2016. Details of the literature search are available online (Additional file 1: Appendix A). Author CY screened papers for eligibility by reading abstracts and, where necessary, the full text. A second reviewer (CH) independently read 25% of the papers and compared her findings with the first author. Disagreements were resolved by discussion. Of the 159/492 (25%) papers independently assessed by the first and second author, four discrepancies were detected; however on closer inspection all of these papers met exclusion criteria and no further papers were assessed by the second author. Reference lists were examined from included papers to identify potentially eligible studies.

### Definition of variables

#### Psychosocial variables

Psychosocial variables were defined as any quantifiable measure of children’s characteristics, and their family and community environments. These were grouped into commonly occurring domains (e.g. socioeconomic status). Domains were further grouped by individual, family and community level. Individual-level domains relate to children’s traits, attitudes or abilities; family-level domains relate to the family/household environment, including parent’s characteristics and relationships with children; community-level domains relate to children’s neighbourhood and broader community, including peer relationships and school-based variables. Domains that were measured in fewer than four papers were not included in this analysis. This arbitrary rule was decided by the authors in order to include domains that were likely to provide sufficient data for comparison and evaluation purposes. The list of domains and their definitions are given below:

### Individual-level domains

#### Optimism

Measured children’s optimistic view of their future and optimistic explanatory styles.

#### Positive attitudes towards school

Measured children’s positive view of school including feelings of school membership.

#### Self-efficacy

Measured children’s belief in their ability to achieve specific goals.

#### Self-esteem

Measured children’s concept of their own self-worth.

#### Identification with white culture

Measured the extent that Indigenous children saw themselves adopting or adapting to White cultural practices. This domain was measured primarily with ethnic identification scales. For example, the Orthogonal Cultural Identification Scale (OCIS) [19] or the Bicultural Ethnic Identity Scale [20].

#### Scholastic ability

Measured children’s academic achievement or general cognitive ability. Grade Point Average (GPA) scores were the most commonly used measure for this domain.

#### Identification with indigenous culture

Measured children’s identification with their own Indigenous culture. This domain was primarily measured with ethnic identification scales (e.g. the OCIS), or by assessing children’s knowledge of their Indigenous culture or language.

#### Substance use

Measured children’s use of illegal drugs and alcohol (tobacco use was not included).

#### Externalising

Measured antisocial, aggressive and oppositional behaviours.

#### Internalising

Measured internalising symptoms including anxiety, depression, withdrawn behaviour and suicidal ideation.

#### Adverse events

Measured children’s exposure to events likely to cause substantial stress (e.g. abuse, neglect) or significant disruption to children’s lives (e.g. the loss of a close family member).

### Family-level domains

*Family cohesion (positive):* Measured the quality of relationships children experienced within their immediate family including measures of family support and positive parenting styles.

#### Low family SES

Measured indices of socio-economic status (SES) including family income, caregiver’s education and occupation, household occupancy level and housing quality/tenure.

#### Atypical family structure

Measured whether children were raised by single caregivers or by family members other than the children’s parents (e.g. aunts, uncles or grandparents).

#### Caregiver’s mental health/behaviour (negative)

Included measures of caregiver’s mental health problems, criminal activity, domestic violence and substance abuse.

#### Family cohesion (negative)

Measured poor relationships children had with their family, and harsh parenting practices.

### Community-level domains

#### Peer support

Measured the presence and quality of prosocial relationships children had with their peers.

#### Community cohesion (negative)

Measured negative elements within the children’s community including violent or criminal activity in neighbourhood or school environments.

#### Discrimination

Measured children’s experiences of racial discrimination.

#### Bullying

Measured whether children had experienced recent bullying.

#### Mental health outcomes

We defined mental health outcomes as any internalising or externalising symptom, and/or measure of positive mental health typically associated with school-aged children. Internalising disorders describe adverse mental health states that are inner-directed, including depression, anxiety, and withdrawal [21]. In contrast, externalising disorders are outer-directed and manifest as maladaptive behavioural problems including antisocial, oppositional and aggressive behaviour [22].

Positive mental health outcomes included measures of self-esteem, positive affect and resilience. Resilience is commonly defined as positive adaption in the presence of adversity [23]. Studies that measured associations between psychosocial variables and mental health outcomes in conjunction with elevated levels of adversity were deemed to measure ‘resilient’ mental health. For example, Hopkins et al. [24] divided a sample of Australian Aboriginal children into ‘low’ and ‘high’ risk groups based on the number of adversities experienced. Children in the high-risk group who showed good mental health outcomes (as measured by the Strengths and Difficulties Questionnaire) [25] were considered resilient. Studies that did not include a measure of adversity or a validated resilience scale were not deemed to measure resilience. A separate summary of the psychosocial variables that were associated with resilient mental health is given in the results.

Mental health measures that combined internalising, externalising or positive mental health outcomes were described as ‘Global’ measures of mental health. For example, the Strength and Difficulties Questionnaire uses measures of ‘conduct problems’ (externalising), ‘emotional symptoms’ (internalising) and ‘prosocial behaviour’ (positive mental health) to calculate a global measure of children’s mental health.

In order to assess comorbidity between mental health outcomes, externalising, internalising and self-esteem constitute both predictor variables (domains) and outcomes (mental health) in this review.

### Data extraction strategy

Bivariate and multivariable analyses of a domain’s association with mental health were extracted from each study, including the statistic used, the magnitude and direction of association, the *p*-value and the confidence interval (where given). When path analysis was employed, only associations from the best fitting model were included. Similarly, when multiple statistical models progressively introduced confounders, only statistics from the final modal were included. Longitudinal and cross-sectional data were both included. Interactions were not recorded; however, because the construct of resilience can be observed through statistical interactions between levels of adversity and other predictor variables, interactions that were deemed to measure resilient mental health were included. When multiple papers reported results from the same study, variables measuring the same domain were treated as belonging to a single study.

### Data synthesis and presentation

The aim was to determine the associations between psychosocial variables and childhood mental health outcomes. Due to the considerable heterogeneity in how these variables were conceptualised and measured, and in the statistical methods employed to assess relationships, calculation of summary estimators (meta-analysis) was neither possible nor appropriate. Instead, a two-stage process was used to assess the strength of association between psychosocial variables and mental health. The first stage involved making an overall judgement whether an individual study provided evidence for an association between a domain and: good mental health, poor mental health, or showed a negligible or inconsistent association. The second stage involved assessing the quality of evidence associating each domain with mental health, as measured by multiple studies, using the Grades of Recommendation, Assessment, Development, and Evaluation (GRADE) [26].

#### Individual studies

Each study was independently assessed by two authors (CY, CH) to ascertain whether it provided evidence for an association between a psychosocial domain and: good mental health, poor mental health, or a negligible or inconsistent association. When only one association between a psychosocial domain variable and a mental health outcome was reported in a single study, statistical significance was used to determine evidence for an association. When domains were measured by more than one psychosocial variable and/or multiple mental health outcomes were used within a single study; the number of statistically significant associations, the magnitude and direction of effects and the number of comparisons were all considered before making a judgement regarding an association. Measures of both positive (e.g. self-esteem) and negative (e.g. depression) mental health were considered together in order to determine the overall association between domain variables and mental health. Disagreements were resolved via discussion.

### Study quality assessment

We used the Grades of Recommendation, Assessment, Development, and Evaluation (GRADE) guidelines to rate the quality of evidence within each domain. The GRADE guidelines rate evidence as being ‘very low’, ‘low’, ‘moderate’ or ‘high’ depending on four categories of investigation: risk of bias, inconsistency, indirectness, and if reasons to rate up the strength of evidence exist. The GRADE category of ‘Imprecision’ was not assessed given the relatively small number of studies that reported confidence intervals. The GRADE category of ‘Indirectness’ was also not assessed given that relevant inclusion criterion were matched directly to the research question. Observational studies start at ‘low’ quality and could be rated up or down depending on the quality of evidence. In accordance with the GRADE recommendations, domains that had been rated down for any reason were not eligible to be rated up. Two authors (CY, CH) independently assessed all elements of the GRADE evidence profile, discrepancies were resolved by discussion.

#### Risk of bias

Risk of bias was first assessed in individual papers using the Newcastle-Ottawa Scale (NOS) adapted for cross-sectional studies [27]. This scale measures potential sources of bias on a 10-point scale. Risk of bias is deemed to be present if the sample size is not justified or unsatisfactory [28], if the sample is unrepresentative of the target population, if inappropriate or un-validated measurement tools have been used, if theoretically important variables were not controlled for (socioeconomic status, and age and gender), and if inappropriate or unclear statistical tests were employed. We set the following criteria for judging risk of bias: 9–10 points = low risk; 7–8 points = medium risk; ≤6 points = high risk. Domains that included a majority of high risk studies were considered to be at serious risk of bias and were rated down.

#### Inconsistency

Inconsistency was deemed to be present when large differences between point estimates and/or confidence interval ranges were observed among studies that measured the same psychosocial domain. Domains were always rated as inconsistent if different studies measuring the same domain produced statistically significant but conflicting associations with mental health outcomes (note: this did not include negligible associations).

#### Rating up the quality of evidence

Provided that there were no reasons to rate evidence down, the quality of evidence for each domain could be rated up if: the majority of studies reported medium or large effect sizes, if a dose-gradient effect was observed, or if the majority of studies controlled for confounding variables that could plausibly reduce the magnitude of the effect. We followed conventional rules of thumb for effect sizes [29] and deemed medium effect sizes as: Cohen’s *d* = .5, zero-order correlation coefficient *r* = |.3|, and odds ratios = 2 or .5; large effect sizes were defined as Cohen’s *d* = .8, zero-order correlation coefficient *r* = |.5|, and odds ratios = 5 or.2. All other statistics were interpreted within the context of the study.

Using the above heuristics two researchers (CY, CH) independently appraised the effect sizes reported in each study. Effect sizes were rated as being ‘small’, ‘medium’, ‘large’, ‘negligible’ or ‘inconsistent’. When more than one statistic was reported, a summary of the range of effect sizes was recorded, outliers were excluded. Using the same method, a qualitative summary of the range of effect sizes, *per domain*, was made by the researchers, disagreements were resolved by discussion.

For example, a study by Whitbeck et al. [30] investigated substance use among American Indian children. In this case the domain, ‘substance use’ is indicated by three variables: “alcohol problems”, “alcohol abuse” and “number of substances used in the past month”. Mental health was indicated by measures of withdrawal, somatic complaints and anxiety/depression (all symptoms of internalising). This study provided three independent variables and three dependent variables, yielding nine associations between the domain ‘substance use’ and mental health. The variable “number of substances used in the past month” was found to be significantly correlated with mental health variables: “somatic symptoms” and “anxiety/depression” (*r*’s = .16 and .27, respectively). All other correlations were positive but non-significant. Given the absence of conflicting evidence, and the two significant correlations, this paper is deemed to have provided evidence of an association between the domain ‘substance use’ and poor mental health.

After appraising all other studies measuring the domain ‘substance use’, 8/9 studies measuring this domain were deemed to provide evidence for an association with poor mental health. Using the GRADE guidelines the quality of evidence was rated up from ‘low’ to ‘moderate’ due to the majority of studies that adjusted for confounding factors and the absence of any reason to rate down.

## Results

### Review statistics

**Fig. 1:**
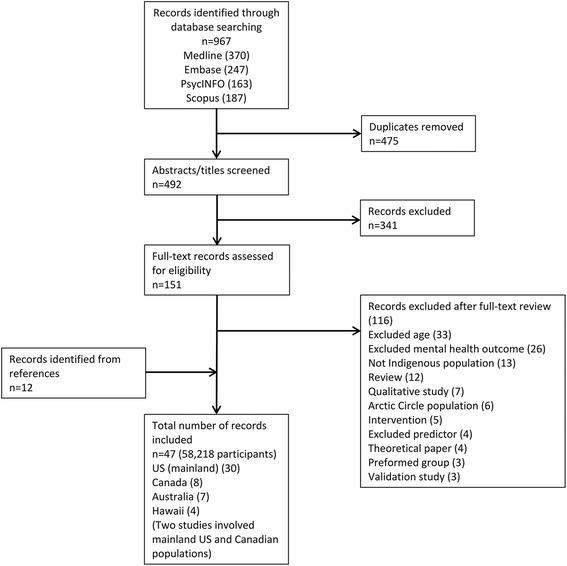
Search results

Forty-seven papers were included in the review. Figure 1 presents the results of the literature search.

The majority of papers reported on studies conducted in the US (mainland; 30 papers, 64%) with Native American samples, 8 papers (17%) involved Indigenous Canadian samples (two papers assessed both US mainland and Canadian Indigenous samples), 7 papers (15%) involved Indigenous Australian children, and 4 (9%) papers involved Indigenous Hawaiian children. No studies from New Zealand met inclusion criteria. All studies were observational; 39 papers (83%) used a cross-sectional design, 8 (17%) used a longitudinal design or a mixture of longitudinal and cross-sectional designs. Participants’ ages ranged from 4 to 20 years. Most studies included children aged between 11 and 18 years (i.e. middle and/or high school-aged children). Sample sizes ranged from 65 to 13,454 participants. Measures of negative mental health outcomes were the most commonly assessed, measured in 41 (87%) papers. Internalising symptoms were measured in 27 papers (57%), externalising symptoms were measured in 14 papers (30%), global measures of mental health were measured in 14 papers (30%), and positive mental health was measured in 13 papers (28%). Domains that appeared in the search but were measured in fewer than four papers included: physical health, historical loss, religious involvement, level of isolation, social skills and self-regulation. The number of publications was seen to increase over time with half of the papers published between 2011 and January 2016 (the last five years of the review’s 20-year timeframe).

Individual-level domain variables were reported in 40 papers (85%), family-level domain variables were measured in 25 papers (53%) and community-level domain variables were measured in 22 papers (47%). The median number of associations between a single psychosocial domain and mental health outcome per paper was two (interquartile range: 3). Table 1 provides a summary of the included papers.

**Table 1:** Study characteristics

Region	Study	Sample size	Male (%)	Age (range or *mean*) or school grade	Mental health outcome	Mental health measure
US (mainland)						
	Costello [35], 1997	323	53	9–13	Symptoms of child/adolescent psychiatric disorders	CAPA
	Federman [36] 1997	431	Not reported	9–15	Symptoms of child/adolescent psychiatric disorders	CAPA
	Cummins [45], 1999	13,454	49	*14.5*	Positive mental health	Emotional Health scale (bespoke measure)
	Fisher [66], 1999	112	46	*14.82*	Psychopathological behaviour	CBCL
	Wall [72], 2000	96	52	8–13	Internalising and externalising symptoms	CBCL
	Whitbeck [30], 2001	195	54	9–16	Internalising symptoms	YSR
	Rieckmann [39], 2004	332	41	14–20	Depression	CDI, DSM-IV, MMPI
	Bearinger [40], 2005	569	48	9–15	Violence	Bespoke measure
	Newman [52], 2005	96	47	12–15	Internalising symptoms, positive mental health	SAS, SMFQ, RSE, PANAS-X, YSR, SEQ, FES
	La Fromboise [60], 2006	212	54	10–15	Positive mental health	Bespoke measure
	Silmere [67], 2006	401	45	*15.6*	Positive mental health	DIS-IV, YSR, CIS
	Whitesell [70], 2006	1252	48	14–17	Self-esteem	RSE
	Jones [46], 2007	137	47	14–19	Self-esteem, depression	RSE, CES-D
	Stiffman [62], 2007	385	Not reported	12–19	Behaviour and emotional problems	YSR
	Stiffman [47], 2007	401	Not reported	12–19	Depression, conduct disorder	YSR, CIS
	Scott [49], 2008	112	53	13–19	Depressive symptoms	IDD
	Hamill [58], 2009	151	54	7-12th grade	Depressive symptoms	CDI
	Albright [54], 2010	114	47	11–15	Hopelessness	HSC
	La Fromboise [55], 2010	438	46	Adolescents	Hopelessness	BHS
	Galliher [56], 2011	137	49	14–19	Self-esteem, social functioning	CASAFS, RSE
	Scott [50], 2012	198	46	5-8th grade	Depressive symptoms	CDI
	Stumblingbear-Riddle [48], 2012	196	42	14–18	Self esteem	TECSES
	Mileviciute [41], 2013	93	51	Grades 5–8	Depressive symptoms	CDI
	Mileviciute [51], 2014	146	36	13–18	Depressive symptoms, externalising problems	CDI, YSR
	Smokowski [42], 2014	1358	49	13.4	Internalising and externalising symptoms, self-esteem	SSP, YSR, RSE
	Bell [74], 2014	79	41	11–18	Depressive symptoms, self-esteem	CES-DC, RSE
	Tyser [43], 2014	164	47	Grades 5–12	Depressive symptoms	CDI
	Brokie [68], 2015	132	49	15–19	Depression and PTSD symptoms	BDI-IA, Short Screen for PTSD
US (mainland) and Canada						
	Hartshorn [65], 2012	692	50	10–12 at first wave	Aggression	DSM-IV
	Whitbeck [73], 2006	656	50	9–13	Childhood mental disorders	DISC-R
Canada						
	Mykota [57], 2006	480	51	6–18	Psychosocial functioning	BRP-2
	Flanagan [61], 2011	65	58	11–19	Internalising and externalising symptoms	T-CRS, CDI, RCMAS-2, peer report
	Lemstra [53], 2011	204	44	5–8 grade	Depressed mood	CES-D
	Lemstra [75], 2011	204	44	10–16	Depressed mood	CES-D
	Ames [44], 2013	283	48	*12*	Depressive symptoms, self-esteem	CES-D, SDQ-2
	Kaspar [71], 2013	12,366	51	6–14	Psychological or nervous difficulties	Clinical diagnosis
Australia						
	Silburn [31], 2007	1073	Not reported	12–17	Clinically significant emotional and behavioural problems	SDQ
	Priest [63], 2011	345	47	16–20	Social and emotional wellbeing	Strong Souls Survey
	Zubrick [32], 2011	5289	Not reported	0–17	Clinically significant emotional and behavioural problems	SDQ
	Shepherd [33], 2012	3993	51	4–17	Clinically significant emotional and behavioural difficulties	SDQ
	Askew [69], 2013	344	52	7.3	Child’s behaviour	Parent report
	Hopkins [34], 2013	674	50	12–17	Clinically significant emotional and behavioural difficulties	SDQ
	Hopkins [24], 2014	1021	50	12–17	Clinically significant emotional and behavioural difficulties	SDQ
Hawaii						
	Makini [64], 1996	1819	45	Grades 9 to 12	Internalising and externalising symptoms	CES-D, STAI, BADS
	Goebert [37], 2000	2634	Not reported	Grades 9 to 12	Internalising and externalising symptoms	CES-D, STAI, BADS
	Carlton [38], 2006	1173	46	Grades 9–12	Internalising and externalising symptoms	CES-D, STAI, BADS
	Hishinuma [59], 2012	3189	46	Grades 9–12	Depression	CES-D

*BADS* Braver Aggression Detection Scale; *BDI*-*IA* amended Beck Depression Inventory; *BHS* Beck Hopelessness Scale; *BRP*-*2* Behaviour Rating Profile-2nd Edition; *CAPA* Child and Adolescent Psychiatric Assessment; *CASAFS* Child and Adolescent Social and Adaptive Functioning Scale; *CBCL* Child Behaviour Checklist; *CDI* Children’s Depression Inventory; *CES*-*D* Centre for Epidemiology Studies-Depression; *CIS* Columbia Impairment Scale; *DBD* Disruptive Behaviour Disorders Rating Scale; *DIS*-*IV* National Institute for Mental Health’s Diagnostic Interview Schedule; *DISC*-*R* Diagnostic Interview Schedule for Children-Revised; *DSM*-*IV* Diagnostic and Statistical Manual of Mental Disorders-Fourth Edition; *FES* Family Environment Scale; *HSC* The Hopelessness Scale for Children; *IDD* Inventory to Diagnose Depression; *MMPI* Minnesota Multiphasic Personality Inventory; *PANAS*-*X* Positive and Negative Affect Schedule; *RCMAS*-*2* Revised Children’s Manifest Anxiety Scale; *RSE* Rosenberg Self-Esteem Scale; *SAS*-*A* Social Anxiety Scale for Adolescents; *SDQ* Strengths and Difficulties Questionnaire; *SDQ*-*2* Marsh’s Self-Description Questionnaire; *SEQ* Social Experiences Questionnaire; *SMFQ* Short Mood and Feelings Questionnaire; *SSP* School Success Profile; *STAI* Spielberger State-Trait Anxiety Inventory; *T*-*CRS* Teacher-Child Rating Scale; *TECSES* Tri-Ethnic Center’s Self Esteem Scale; *YSR* Youth Self-Report

### Study quality assessment

**Fig. 2:**
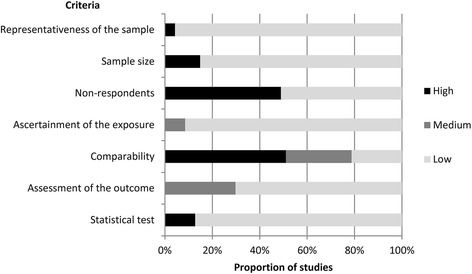
Risk of bias

Figure 2 presents the results of the Newcastle-Ottawa scale assessment. Scores ranged from 4 to 10 (median: 7). 12 papers (26%) were judged to have low risk of bias, 21 papers (45%) were judged to have medium risk of bias, and 14 papers (30%) were judged to have high risk of bias. 23 papers (49%) failed to report information regarding non-respondents or reported a response rate that was less than 75%, 37 papers (79%) failed to control for age and gender, and/or any socioeconomic variables, though most papers (36, 77%) controlled for at least one other variable, 14 papers (30%) used measures of mental health that were not culturally validated.

### Evidence of effectiveness

Tables 2, 3 and 4 present the GRADE evidence profile for individual, family and community level domains.

**Table 2:** GRADE evidence profile for individual-level domains

Domain	Number of studies	Risk of bias	Inconsistency	Effect size	Quality	Comments
Optimism	7	No serious risk	No serious inconsistency	Small-medium	Moderate	Rated up due to control of confounding factors
Positive attitudes towards school	5	No serious risk	No serious inconsistency	Small-medium	Low	Studies from the US (mainland) only
Self-efficacy	4	No serious risk	No serious inconsistency	Small-medium	Moderate	Rated up due to control of confounding factors Studies from the US (mainland) only
Self-esteem	9	No serious risk	No serious inconsistency	Small-large	Moderate	Rated up due to evidence of a dose-gradient effect
Identification with White culture	6	No serious risk	No serious inconsistency	Negligible-Small	Low	Studies from the US (mainland) only
Scholastic ability	8	No serious risk	Serious inconsistency	Inconsistent	Very low	Rated down due to inconsistent findings
Identification with Indigenous culture	20	No serious risk	Serious inconsistency	Inconsistent	Very low	Rated down due to inconsistent findings
Substance use	9	No serious risk	No serious inconsistency	Small-Large	Moderate	Rated up due to control of confounding factors
Externalising	7	Serious risk of bias	No serious inconsistency	Medium	Very low	Rated down due to serious risk of bias
Internalising	7	No serious risk	No serious inconsistency	Medium-Large	Moderate	Rated up due to medium-large effect sizes
Adverse events	8	No serious risk	No serious inconsistency	Medium-large	High	Rated up due to medium-large effect sizes, a dose-gradient effect and satisfactory control of confounding factors

*GRADE* Grades of Recommendation, Assessment, Development, and Evaluation

**Table 3:** GRADE evidence profile for family-level domains

Domain	Number of studies	Risk of bias	Inconsistency	Effect size	Quality	Comments
Family cohesion (positive)	12	No serious risk	No serious inconsistency	Small-large	Moderate	Rated up due to evidence of a dose-gradient effect
Low family SES	8	No serious risk	Serious inconsistency	Inconsistent	Very low	Rated down due to inconsistent findings
Atypical family structure	6	No serious risk	No serious inconsistency	Negligible-small	Moderate	Rated up due to control of confounding factors
Caregiver mental health/behaviour (negative)	8	No serious risk	No serious inconsistency	Small-large	Moderate	Rated up due to control of confounding factors
Family cohesion (negative)	6	No serious risk	No serious inconsistency	Medium-large	High	Rated up due to medium-large effect sizes and a dose-gradient effect

*GRADE* Grades of Recommendation, Assessment, Development, and Evaluation; *SES* Socioeconomic Status

**Table 4:** GRADE evidence profile for community-level domains

Domain	Number of studies	Risk of bias	Inconsistency	Effect size	Quality	Comments
Peer support	5	No serious risk	No serious inconsistency	Small-Medium	Low	
Community cohesion (negative)	4	No serious risk	Serious inconsistency	Negligible-Large	Very low	Rated down due to inconsistent findings Studies from US (mainland) and Canada only
Discrimination	8	No serious risk	No serious inconsistency	Small-Medium	Moderate	Rated up due control of confounding variables
Bullying	4	No serious risk	No serious inconsistency	Small-Large	Low	Studies from US (mainland) and Canada only

*GRADE* Grades of Recommendation, Assessment, Development, and Evaluation

**Fig. 3:**
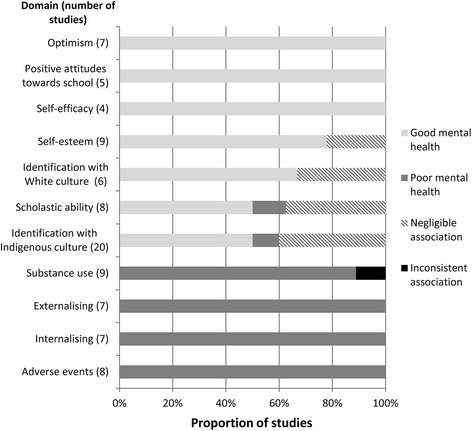
Individual-level associations

**Fig. 4:**
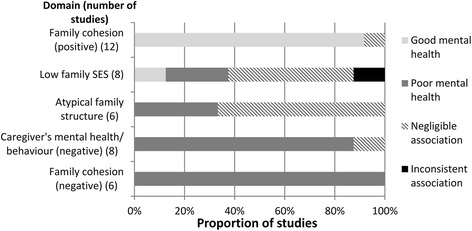
Family-level associations

**Fig. 5:**
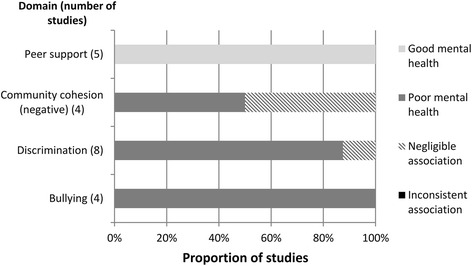
Community-level associations

Figures 3, 4 and 5 show the number of studies that measured each individual, family, and community-level domain’s association with mental health, respectively, and the proportion of studies, within each domain, associated with good mental health, poor mental health, or those that showed a negligible or inconsistent association. Five papers from Australia used data from same large-scale study (Western Australian Aboriginal Child Health Survey) [24, 31–34], two papers from the US (mainland) used data from the same study (Great Smokey Mountains Study) [35, 36], and two papers from Hawaii used data from the same study (Native Hawaiian Mental Health Research Development Program) [37, 38]. To avoid overinflating the number of associations, these papers were treated as a single study when they measured the same domain.

#### Individual-level domains

##### Optimism

Optimism was associated with better mental health outcomes in all studies (7/7) that measured this domain [38–44]. Optimism was negatively associated with internalising symptoms in all six studies that measure this outcome.

##### Positive attitudes towards school

Positive attitudes towards school were consistently associated with better mental health outcomes in all studies (5/5) that measured this domain [40, 45–48]. This domain was only assessed in studies conducted in the US (mainland).

##### Self-efficacy

Self-efficacy was associated with good mental health in all studies (4/4) that measured this domain [43, 49–51]. Using a cross-sequential longitudinal design one study found increases in self-efficacy predicted decreases in depressive symptoms over a three-year period [50]. This domain was only assessed in studies conducted in the US (mainland).

##### Self-esteem

High self-esteem was associated with better mental health outcomes in 7/9 (78%) of the studies that measured this domain [24, 42, 44–46, 52, 53]. One study of Aboriginal Australian children showed a dose-gradient effect linking higher levels of self-esteem to greater odds of positive psychosocial functioning [24]. Medium to high negative correlations between self-esteem and depressive symptoms were reported (correlation coefficients ranged from −.26 to −.71).

##### Identification with white culture

Greater identification with White culture was significantly associated with better mental health outcomes in 4/6 (67%) studies [46, 54–56]. This domain was only assessed in studies conducted in the US (mainland).

##### Scholastic ability

Greater scholastic ability was significantly associated with better mental health outcomes in 4/8 (50%) studies [38, 43, 48, 57], however this domain’s relationship with mental health was inconsistent with one study showing that higher GPA was significantly associated with increased depressive symptoms [58]. The highest quality study, a cohort-sequential design, provided evidence that depression negatively affects scholastic ability, not the other way around [59].

##### Identification with indigenous culture

Children’s identification with their own Indigenous culture was found to be significantly associated with better mental health outcomes in 10/20 (50%) studies [39, 42, 43, 46, 48, 52, 55, 56, 60, 61]. Conversely, two studies conducted in the US (mainland) and Hawaii found this domain to be associated with poor mental health [38, 47]. Identification with Indigenous culture appeared more strongly associated with measures of positive mental health (i.e. self-esteem, significantly associated in 6/9 studies) than measures of negative mental health (significantly negatively associated in 5/14 studies).

##### Substance use

Substance use was associated with poorer mental health in 8/9 (88.9%) studies [30, 36, 40, 46, 51, 62–64]. Substance use was consistently associated with externalising and global measures of poor mental health (5/5 studies) [36, 40, 51, 62, 63], but was less consistently associated with depressive symptoms (4/8 studies) [30, 46, 63, 64].

##### Externalising

All studies (7/7) that measured externalising symptoms found a positive association between this domain and other negative mental health outcomes [30, 46, 51, 52, 61, 64, 65]. Externalising symptoms were associated with symptoms of depression in 5/5 studies [30, 46, 51, 52, 64], with other symptoms of externalising in 2/2 studies [61, 65], and negatively associated with positive mental health in 1/2 studies [46]. The evidence for externalising was rated down due to 4/7 (57%) studies having a high risk of bias [51, 52, 61, 64].

##### Internalising

All studies (7/7) that measured internalising symptoms found a positive association between this domain and other negative mental health outcomes [30, 40, 44, 45, 51, 62, 64]. Internalising symptoms were associated with symptoms of externalising symptoms in 3/3 studies [40, 51, 64], with global measures of poor mental health in 2/2 studies [45, 62], with other internalising symptoms in 2/2 studies [30, 64], and were negatively associated with positive mental health in one study [44].

##### Adverse events

Children’s experience of adverse events was associated with poorer mental health in all (9/9) papers that measured this domain [31, 32, 41, 53, 62, 66–69]. Two papers used data from the same study [31, 32], therefore, 8/8 studies were ultimately recorded as showing an association between adverse events and mental health. The evidence linking adverse events and negative mental health included large effect sizes (maximum odds ratio: 8.9; Cohen’s *d*: 1.55), and two studies that reported a dose-gradient response between the number of adversities and prevalence of poor mental health [31, 68].

#### Family-level domains

##### Family cohesion (positive)

This domain was significantly associated with better mental health outcomes in 12/13 papers [37, 38, 40, 45, 48, 53, 60, 62, 66, 67, 70, 71]. Two papers used data from the same study [37, 38], therefore, 11/12 (92%) studies were ultimately recorded as showing an association between positive family cohesion and mental health.

##### Low family SES

Low family SES was significantly associated with poor mental health in 4/11 papers [33, 34, 37, 65]. Four papers using data from the same study found an inconsistent relationship with mental health [24, 31, 33, 34], with two papers showing low SES was associated with less odds of emotional and behavioural problems [24, 31], and two further papers reporting that low SES was associated with increased odds of emotional or behavioural problems [33, 34]. These four papers were treated as one study showing inconsistent outcomes. Therefore, 2/8 (25%) studies were ultimately recorded as showing an association between low family SES and poor mental health [37, 65]. A Canadian study found that children of caregivers who had some postsecondary education were more likely to have a diagnosed psychological or nervous condition than those who did not have any post-secondary education [71]. The remaining studies found negligible associations.

##### Atypical family structure

Atypical family structure was associated with poor mental health in 4/8 papers [31, 32, 34, 37]. Three papers used data from the same study [31, 32, 34], therefore, 2/6 (33%) studies were ultimately recorded as showing an association between atypical family structure and poor mental health.

##### Caregiver’s mental health/behaviour (negative)

This domain was associated with poor mental health outcomes in 9/10 papers [24, 31, 34, 35, 37, 40, 68, 72, 73]. Three papers used data from the same study [24, 31, 34], therefore, 7/8 (88%) studies were recorded as showing an association between caregiver’s negative mental health or behaviour and children’s mental health. Violence between caregivers, and caregiver’s anti-social behaviour produced the strongest association with poor mental health (bivariate odds ratios: 5.6 and 7.1, respectively) [40, 68].

##### Family cohesion (negative)

Negative family cohesion was associated with poor mental health in 7/7papers [31, 34, 52, 53, 62, 67, 68]. Two papers used data from the same study [31, 34], therefore, 6/6 studies were recorded as showing an association between this domain and poor mental health. Effect sizes were medium to large in all studies that reported them (one study did not report effect sizes [67]). Children who stated that they rarely had someone who showed them love and affection [53] or who reported more family conflict [52] showed the strongest associations with poor mental health (odds ratio: 4.8, correlation coefficient: .55, respectively).

#### Community-level domains

##### Peer support

All studies (5/5) that investigated peer support found an association between this domain and better mental health outcomes [34, 40, 48, 52, 71].

##### Community cohesion (negative)

Negative community cohesion was associated with poor mental health in 2/4 (50%) studies [62, 67]. Only studies from the US (mainland) and Canada assessed this domain.

##### Discrimination

Discrimination was observed to be associated with poor mental health in 8/9 papers [24, 30, 56, 60, 63, 65, 67, 68]. Two papers used data from the same study [24, 63], therefore, 7/8 (88%) studies were recorded as showing an association between discrimination and mental health. Using an auto-regressive cross-lagged path design, a study of Native American and Canadian Indigenous groups concluded that discrimination caused subsequent aggression and not the other way around [65].

##### Bullying

Bullying was associated with poor mental health in 4/4 papers [52, 53, 74, 75]. Only studies from US (mainland) and Canada assessed this domain.

#### Resilience

Five studies provided a quantitative measure of both adversity and mental health, fitting the inclusion criteria for ‘resilience’. These included one Australian, one Hawaiian, and three studies from the US (Mainland) [24, 37, 41, 56, 60].

Of the three studies conducted with Native American youths, resilient mental health was significantly associated with identification with Indigenous culture, maternal warmth, not experiencing discrimination, optimistic explanatory styles, and identification with White culture (females only) [41, 56, 60]. One Australian study found resilient Aboriginal youths were more likely to have higher self-esteem, be less likely to be involved in fights, have a prosocial friend, and be less likely to live in the top 50% of neighbourhoods, as rated by an index of neighbourhood SES [24]. Identification with Aboriginal culture was not found to be significantly related to resilience in this study. A study of Hawaiian youths found that family support lessened the likelihood of internalising symptoms in children experiencing multiple family adversities [37].

## Discussion

Any discussion of Indigenous disadvantage must first acknowledge the longstanding inequalities many Indigenous people continue to face, and the subsequent influence this can have on all aspects of their lives [76]. Within this context, many risk factors may also be considered as downstream effects of historical trauma.

Moderate to high level evidence exists for associations between a number of psychosocial domains and the mental health of Indigenous children living in high income countries. Of these, domains associated with better mental health outcomes included: children’s positive cohesion with their family, higher self-efficacy, self-esteem and optimism. Domains associated with poorer mental health outcomes included: caregiver’s negative mental health/behaviour, discrimination, co-morbid internalising symptoms, and substance use. The highest quality evidence indicated that negative family cohesion and children’s experiences of adversity predicted poorer mental health, with both domains consistently producing medium to large effect sizes. Studies focused on adolescents, and predominantly measured symptoms of poor mental health. Despite a growing body of work in this area, the amount of research that investigates the aetiology of Indigenous children’s mental health appears small relative to need.

The association between children’s identification with their Indigenous culture and mental health was the most commonly assessed association, reflecting the importance that community-led research and Indigenous mental health initiatives place on this relationship [77–79]. This domain generally predicted better mental health outcomes however evidence for this association was inconsistent. Children’s identification with their Indigenous culture was seen to be a factor that promoted resilient mental health in a sample of American Indian children [60], indicating that cultural identification may be a protective factor when adversity is present, however this finding was not replicated in Australian Aboriginal children [24]. Differences in the way cultural constructs are operationalized, and difficulties measuring this construct have been previously reported and may account for the heterogeneous findings [80, 81]. Research that can identify the specific processes that allow Indigenous children’s identification with their culture and with White culture to protect against poor mental health is suggested as an area for more detailed investigation.

In contrast, relationships between individual-level psychological factors and mental health outcomes appeared more stable, indicating the importance of fostering optimistic attitudes, self-esteem and self-efficacy in Indigenous young people. These results suggest that community initiatives that seek to empower Indigenous children are likely to prevent some occurrences of poor mental health.

Our results are consistent with findings from non-Indigenous research that show the important influence the familial environment has on children’s mental health [82–85]. Of the 18 studies that measured family cohesion, 17 were judged to provide evidence for an association with mental health, including medium to large effect sizes reported in studies from all regions. Moreover, our results illustrate the clear correlation family cohesion has with mental health outcomes: positive cohesion predicted better mental health, whereas negative cohesion predicted worse mental health. Negative caregiver behaviour, such as criminal activity or the presence of domestic violence and poor mental health was also robustly associated with poorer mental health outcomes in children, as was the domain ‘adverse events’, which often included adversities that were directly related to parent’s behaviour (e.g. neglect). Taken together, these results provide strong evidence that the quality of familial relationships and the presence of stable, supportive family environments are highly predictive of the mental health of Indigenous children.

Low family SES and atypical family structures appeared less consistently associated with mental health. There is a large body of evidence that shows SES is linked to children’s mental health in non-Indigenous populations [86–88]. While the results provide some evidence in support of this research, socioeconomic and family structure factors do not appear to be as reliable predictors of mental health as the types of relationships and stability caregivers are able to provide for Indigenous children. It is possible that limited variation in Indigenous family’s SES, due to ongoing disadvantage, reduced the strength of associations with mental health, resulting in negligible or weak associations. Additionally, variation in the way SES variables were measured may also account for inconsistencies in the results.

At the community level, experiences of discrimination were consistently associated with poor mental health, including evidence from a longitudinal study that suggested a causal relationship with aggressive behaviour [65], however, effect sizes were small to medium. This magnitude of effect is consistent with a recent meta-analysis that found an overall zero-order correlation of −.20 (95% CI: −.22 to −.17) between perceived discrimination (predominantly racial) and mental health in adults [89]. We note that the effect sizes reported in this review refer only to *explicit* discrimination and are not necessarily reflective of the impact of implicit discriminatory attitudes/behaviours, as well as the historical effects of systemic racism [90].

Despite the growing call from Indigenous groups for more strengths-based research [91, 92], we found that a comparatively small amount of studies measured positive mental health outcomes, including studies that were specifically designed to assess resilience. Of these, significant associations were identified at the individual, family and community level, supporting common theoretical frameworks that define resilience as a combination of proximal and distal influences [93]. ‘Positive family cohesion’ was the only domain significantly associated with resilience in more than one study.

### Limitations

This review contains a number of limitations. The heterogeneous manner in which both independent and dependent variables were conceptualised and measured prevented a more fine-grained analysis from being performed, and meant qualitative judgements of quantitative data were employed, potentially introducing bias. This review is vulnerable to publication bias that may result in an overestimate of the number of studies that show significant associations between psychosocial variables and mental health. Most studies were cross-sectional and therefore the results may not be indicative of causal relationships; it is also possible that a bi-directional or reverse causation process may underlie associations. Given similarities between the samples (e.g. socioeconomic status), and that much of the data was self-report, this review may also incur common method bias. Using statistical significance as a primary indicator of an association is problematic as studies that use large samples or employed multiple comparisons are more likely to report significant results. It is therefore likely that this method increased the chance of making a type I error and potentially contributed to a ‘best case’ scenario for detecting associations. Further, we acknowledge that the reliance on arbitrary *p* value thresholds has been widely criticised [94, 95]. We believe the inclusion of the GRADE evidence table and reporting effect sizes help to provide a more thorough description of associations that is not based on *p* values alone. Most studies were conducted in the US (mainland) restricting the generalizability of some domains to other Indigenous groups, similarly some domains were only measured in a small number of studies, this is most notable at the community level. Finally, it is possible that Western ideas and measures of psychopathology do not adequately map onto Indigenous concepts of mental health [96]. Given that the majority of studies used culturally validated measurement tools (measuring both risk/protective factors and mental health outcomes) we are confident that Indigenous concepts of mental health were, for the most part, adequately measured.

## Conclusions

This review highlights several important implications for policy makers, clinicians and Indigenous health researchers. Indigenous children’s family environment appeared a strong universal risk or protective factor for mental health outcomes and comprises a clear target for greater initiatives to promote mental health. Indigenous parents face a number of well-documented stressors that can lead to poor family environments [97, 98]. Further, they face significant cultural and socioeconomic barriers that can prevent them from seeking and receiving adequate health services [99, 100]. While there are programs in place to support caregivers of Indigenous children, given the high rates of mental illness, more needs to be done to enable caregiver’s provision of positive, stable parenting for their children in safe, supportive family environments. This review also supports initiatives that seek to foster positive psychological attributes such as children’s self-esteem, and aim to reduce the incidence of substance use and experiences of discrimination. We identified only three studies that employed research methodologies specifically designed to assess the direction of causality [50, 59, 65]. While study designs of this type often require greater resources to conduct, more research designed to assess causality can provide a richer understanding of the aetiology of Indigenous mental health that can, in turn, aid the construction of effective mental health initiatives.

Large disparities between Indigenous and non-Indigenous health are unacceptable in high income countries that have both the resources and the responsibility to address this inequality. The results of this review emphasise important individual, family and community level factors that comprise potential targets for health interventions. In particular, the strong evidence linking positive familial relationships and environments to better mental health outcomes support the design and implementation of more initiatives to strengthen Indigenous families. However, the lack of Indigenous mental health research, including the small number of longitudinal designs and strength-based research does not appear commensurate with the research and health needs of Indigenous communities. Given the disproportionately high rates of Indigenous mental health disorders and youth suicide, there is an urgent need to address this research gap and develop more evidence-based strategies to reduce the burden of poor mental health for Indigenous children and their families.

## Additional file

Additional file 1: Appendix A. Search strategy. (DOCX 12 kb)

## Abbreviations

ADHD: Attention Deficit Hyperactivity Disorder
CANZUS: Canada, Australia, New Zealand and United States
CI: Confidence interval
GPA: Grade Point Average
GRADE: Grades of Recommendation, Assessment, Development, and Evaluation
MOOSE: Meta-analysis of Observational Studies in Epidemiology
NOS: Newcastle-Ottawa Scale
OCIS: Orthogonal Cultural Identification Scale
SES: Socio-Economic Status
SDQ: Strengths and Difficulties Questionnaire
US: United States
