# Supplementary material for: Psychosocial factors associated with the mental health of indigenous children living in high income countries: a systematic review
Source: Int J Equity Health. 2017 Aug 23;16:153. doi: 10.1186/s12939-017-0652-5 (PMC5568067; doi:10.1186/s12939-017-0652-5)
Supplement: Additional file 1: Appendix A. — Search strategy. (DOCX 12 kb) [file 12939_2017_652_MOESM1_ESM.docx]

**Appendix A. Search strategy**

1. indigenous populations

2. first nation.mp.

3. maori.mp.

4. american indian$.mp.

5. native hawiian.mp.

6. metis.mp.

7. native american.mp.

8. aborigin*.mp.

9. Torres Strait Islander?.mp.

10. indigenous.mp.

11. 1 or 2 or 3 or 4 or 5 or 6 or 7 or 8 or 9 or 10 or 11

12. child*.mp.

13. adolesc*.mp.

14. teen$.mp.

15. student*.mp.

16. youth.mp.

17. 12 or 13 or 14 or 15 or 16

18. well being/ or positive psychology/ or "quality of life"/

19. mental disorders/ or mental health/

20. mental health.mp.

21. exp Adolescent Psychiatry/

22. exp Child Psychopathology/ or exp Child Psychiatry/ or exp Child Psychology/

23. exp Anxiety Disorders/ or exp Separation Anxiety/ or exp Anxiety/ or exp Social Anxiety/

24. internali*ing.mp,hw.

25. externali*ing.mp,hw.

26. behavioural.mp,hw.

27. conduct disorder.mp. or exp conduct disorder/

28. oppositional defiant disorder.mp. or exp oppositional defiant disorder/

29. anxi*.mp.

30. depress*.mp.

31. 18 or 19 or 20 or 21 or 22 or 23 or 24 or 25 or 26 or 27 or 28 or 29 or 30

32. exp Psychological Endurance/ or exp Coping Behavior/

33. protective factors/ or *prevention/ or *"resilience (psychological)"/

34. risk factors/ or *causality/ or *psychosocial factors/ or *risk assessment/ or *sociocultural factors/

35. risk factor?.mp.

36. protective factor?.mp.

37. compensatory.tw.

38. determinent.tw.

39. 32 or 33 or 34 or 35 or 36 or 37 or 38

40. 11 and 17 and 31 and 39

41. limit 40 to (peer reviewed journal and human and english language and yr="1995 -2016")
